# Reported reasons for missing data and the interplay with trial setting

**DOI:** 10.1186/1745-6215-16-S2-P101

**Published:** 2015-11-16

**Authors:** Anna Kearney, Naomi Bacon, Anna Rosala-Hallas, Anne Daykin, Ali Heawood, Athene Lane, Jane Blazeby, Mike Clarke, Paula Williamson, Carrol Gamble

**Affiliations:** 1 University of Liverpool, Liverpool, UK; 2 University of Bristol, Bristol, UK; 3 Queen's University, Belfast, UK

## 

Missing data in clinical trials is common. It can reduce trial efficiency and bias the estimate of treatment effect.

Much emphasis is placed on addressing missing data in trial design. However, strategies need to be informed by the reasons for attrition which are likely to vary according to trial context. In order to develop effective interventions to minimise missing data, it is important to not only understand the causes but also their interplay with the trial setting.

We present the reported reasons for missing data within a cohort of 168 randomised trials published in four major journals in 2013 and 36 Health Technology Assessment programme monographs published 2009-2014.

We discuss the frequency of missing data and differential attrition resulting from causes such as withdrawals due to treatment tolerance or efficacy, inability to measure the primary outcome due to intervening outcomes such as death or illness, laboratory or technological problems, missed measurements by clinical staff and failure of patients to attend visits or return measurements. The frequency of Investigator led post randomisation exclusions will also be reviewed, looking at data excluded for reasons such as protocol violations, post randomisation ineligibility, patients not receiving the intervention or poor treatment adherence.

Levels of missing data and associations with trial setting such as clinical speciality, method and location of primary outcome measurement, nature of the intervention and control, mean age of recruited patients, number of trial centres and countries will be presented.

